# Aflatoxin Exposure after Weaning: Solid Food Contaminant Impairs Growth

**Published:** 2004-09

**Authors:** Julia R. Barrett

Given the heat, humidity, and poor storage conditions of many tropical developing nations, mold readily grows in harvested crops such as maize and groundnuts. Such foods are dietary staples in many of these countries, and their consumption can lead to widespread exposure to aflatoxin, a mold toxin produced by *Aspergillus* species that is known to cause liver cancer. Aflatoxin is also associated with impaired growth and immune function in animals, but minimal data exist regarding comparable effects in humans. To examine a potential link more closely, a team of researchers in the United Kingdom and Benin built upon an earlier cross-sectional study that demonstrated impaired growth among West African children with high aflatoxin exposure **[*EHP* 112:1334–1338]**. The researchers now present evidence from a longitudinal study that aflatoxin does impair growth in humans.

Previous studies indicated that aflatoxin exposure is high in West African populations, and dietary exposure begins with the introduction of solid foods at weaning. Maize, in the form of porridge, is often the first solid food given to young children here. To study the effects on growth of probable aflatoxin exposure at a young age, the team recruited 50 children from each of four villages in the West African nation of Benin. The children were 16–37 months old when the study began in February 2001. The children’s mothers were interviewed in February, June, and October to gather information about each child’s diet, health, and other factors. Blood samples collected from the children at each survey point were analyzed for levels of aflatoxin–albumin, a biomarker of recent aflatoxin exposure. Vitamin A and zinc levels also were obtained as markers of nutrition. The children and their mothers were weighed and measured at each survey point.

At the first survey point, the researchers found that levels of aflatoxin–albumin were significantly higher in weaned children than in those still partially breastfeeding. Throughout the study, more children became fully weaned, and the levels of the biomarker increased in these children. More than 98% of the children were positive for aflatoxin–albumin at all three time points. Most exposure was likely due to maize consumption, although eating other foods such as groundnuts may have contributed.

Children with the highest levels of the aflatoxin biomarker grew an average 1.7 centimeters less than those with the lowest levels. Poor nutrition did not appear to be a factor in the reduced growth, as blood concentrations of vitamin A and zinc were not correlated with aflatoxin–albumin levels.

The mechanism by which aflatoxin could affect growth is currently being investigated. Defining aflatoxin’s effects is complicated by confounding dietary variables (including co-contamination of food with additional mycotoxins) and the presence of infection. For example, previous research by this group revealed an association between aflatoxin exposure and reduced levels of protective antibodies in the saliva of Gambian children. The team therefore theorizes that aflatoxin could affect growth by altering mucosal barriers and lowering resistance to intestinal infection.

The group is now conducting research aimed at better understanding such relationships. They suggest that controlling for many confounding factors will require a randomized intervention study in which aflatoxin exposure would be reduced to assess the toxin’s impact on children’s immunity, growth, and disease susceptibility.

## Figures and Tables

**Figure:**
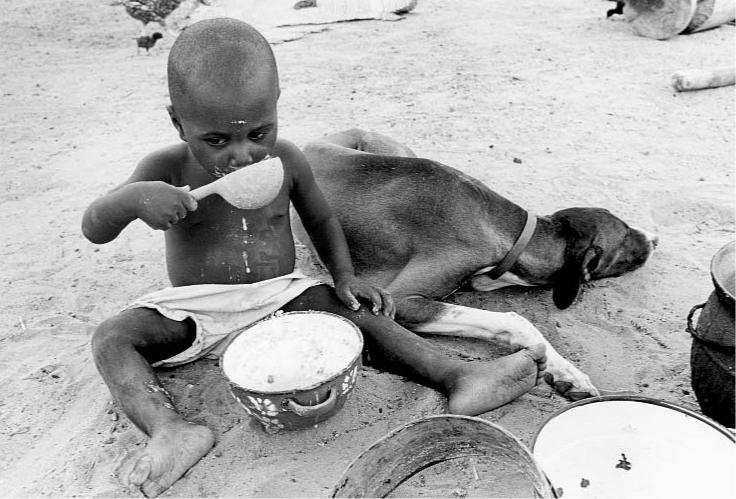
**A somber start.** Maize porridge—a potential source of growth-limiting aflatoxin exposure—is often the first solid food given to West African children such as this boy in Burkina Faso.

